# Landside tritium leakage over through years from Fukushima Dai-ichi nuclear plant and relationship between countermeasures and contaminated water

**DOI:** 10.1038/s41598-020-76964-9

**Published:** 2020-11-16

**Authors:** Katsumi Shozugawa, Mayumi Hori, Thomas. E. Johnson, Naoto Takahata, Yuji Sano, Norbert Kavasi, Sarata K. Sahoo, Motoyuki Matsuo

**Affiliations:** 1 Graduate School of Arts and Sciences, University of Tokyo, 3-8-1 Komaba, Meguro, Tokyo 153-8902 Japan; 2 Department of Environmental and Radiological Health Sciences, Colorado State University, Fort Collins, CO 80523 USA; 3 Atmosphere and Ocean Research Institute (AORI), University of Tokyo, 5-1-5, Kashiwanoha, Kashiwa-shi, Chiba 277-8564 Japan; 4 Institute of Surface-Earth System Science, Tianjin University, Nankai District, Tianjin, 300072 People’s Republic of China; 5 National Institutes for Quantum and Radiological Science and Technology, 4-9-1 Anagawa, Inage-ku, Chiba-shi 263-8555 Japan

**Keywords:** Environmental chemistry, Environmental impact

## Abstract

There has been tritium groundwater leakage to the land side of Fukushima Dai-ichi nuclear power plants since 2013. Groundwater was continuously collected from the end of 2013 to 2019, with an average tritium concentration of approximately 20 Bq/L. Based on tritium data published by Tokyo Electric Power Company Holdings (TEPCO) (17,000 points), the postulated source of the leakage was (1) leaks from a contaminated water tank that occurred from 2013 to 2014, or (2) a leak of tritium that had spread widely over an impermeable layer under the site. Based on our results, sea side and land side tritium leakage monitoring systems should be strengthened.

## Introduction

The Fukushima Dai-ichi Nuclear Power Plant (FDNPP) accident released a large amount of radioactive materials into the environment since 2011. Most were in the gaseous state, released primarily through the atmosphere to the land of eastern Japan and to the north-west Pacific Ocean. The released amount was estimated to be approximately 520 PBq^1^, with radioactive iodine (mainly ^131^I), radioactive cesium (^134^Cs, ^137^Cs), and noble gases such as ^133^Xe accounting for most of the released amount. Tritium (^3^H, T1/2 12.3 y.) was an additional part of the radioactive materials released, but is considered as a “soft”, or low energy, beta emitter. The tritium beta energy is low (max 18.6 keV), and requires large quantities to deliver significant radiation doses, so that the measurement of other nuclear species was prioritized when considering human protection immediately following the accident. Therefore, data on tritium in the environment after the FDNPP accident are still limited in Japan^2,3^.

Tritium in a boiling water reactor is mainly produced by ternary fission. At FDNPP, 8.51 × 10^13^ Bq/month at 1.1 MW operation was produced by ternary fission^4^. Tritium is also produced in reactors by ^10^B(n, 2α)^3^H, ^10^B(n, α)^7^Li, ^7^Li(n, α)^3^H, or ^6^Li(n, α)^3^H, ^2^H(n, γ)^3^H^5^.

Cumulate ^3^H yields in the reactors at FDNPP have been estimated to range from 0.01% to 0.0108%^6,7^. According to estimates made immediately after the accident in 2011, there were reports that the inventory of ^3^H at the time of the accident was 1.81 × 10^13^ Bq^8^, but according to recent reports by Tokyo Electric Power Company Holdings (TEPCO), the inventory of ^3^H immediately after the accident was estimated to be 1.0 × 10^15^ Bq at Unit 1, 1.2 × 10^15^ Bq at Unit 2, and 1.2 × 10^15^ Bq at Unit 3, for a total of 3.4 × 10^15^ Bq^4^. As of March 24, 2016, 7.6 × 10^14^ Bq was in the storage tanks at the FDNPP site, 2.7 × 10^13^ Bq in the reactor building(R/B), and estimated 1.8 × 10^15^ Bq was released outside the reactor or in debris (Table 1)^9,10^.

**Table 1:** Estimates of tritium inventory immediately after the accident.

	TEPCO estimate	Schwantes^8^ estimate
Unit 1	1.0 × 10^15^ Bq	
Unit 2	1.2 × 10^15^ Bq	
Unit 3	1.2 × 10^15^ Bq	
Total	3.4 × 10^15^ Bq	1.81 × 10^13^ Bq

There are three possible pathways for the release of ^3^H from FDNPP to the outside: ocean, atmosphere, and groundwater. Among them, direct releases to the ocean and releases to the atmosphere have been reported in detail.

An estimated 0.1–0.5 PBq of ^3^H flowed into the north Pacific Ocean from the accident^6,11^. Tritium was detected in the north-west Pacific Ocean off the coast of Hirono town, Fukushima Prefecture 1 month after the accident^12^.

Investigation of ^3^H in precipitation may be one of the easiest ways to confirm the release of ^3^H into the atmosphere. The highest tritium concentration in precipitation was estimated 10 days after the accident at 1342 TU (equivalent to 158 Bq/L)^13^. A surface water concentration of ^3^H at 184 (± 2) Bq/L was detected in rice paddy fields at 1.5 km from the FDNPP plant^12^. Since both reports greatly exceeded the natural ^3^H level in Japan (1.1–7.8 TU, equivalent to 0.13–0.92 Bq/L) or 6 TU (equivalent to 0.71 Bq/L)^2,14^, there was no doubt that the ^3^H was from the FDNPP accident. Also, since the samples were collected approximately 1 month after the accident, the ^3^H on the ground most likely originated as precipitation from the atmosphere, not via groundwater.

Leaking of ^3^H through groundwater is difficult to analyze. In this study, we report that ^3^H above natural levels has been detected continuously in groundwater sampled from 2013 to 2019 on land approximately 30 m from the FNDPP site boundary. A key aspect of this study is that the water examined was groundwater, not surface water. To reveal the hydrogeological origin of the groundwater sources, Sr isotope ratio (^87^Sr/^86^Sr) was also measured as a natural tracer of water–rock interaction and ground water mixing patterns^15–18^.

From 2013 to 2019, several countermeasures have been taken at the FDNPP to prevent contaminated groundwater from leaking off site. The relevance will be discussed, including the results of detailed tritium measurements in the water collected inside/outside FDNPP site.

## Results

### Outflow of ^3^H into groundwater from FDNPP

Most of the tritium present in the FDNPP was assumed to have been produced by ternary fission. As long as no re-criticality occurs, no new tritium is produced. However, it is estimated that there is 1.8 × 10^15^ Bq of tritium that has not been identified in the turbine buildings and in contaminated water, in addition to the amount released outside after the accident or the amount in debris^10^. In Japan, the limit for tritium release into the ocean is 6.0 × 10^4^ Bq/L in a typical nuclear facility, but in the case of the FDNPP, 1500 Bq/L is the regulatory limit for tritium effluent^19^. Therefore, over 1.2 × 10^12^ L of water would be required for dilution.

**Figure 1:**
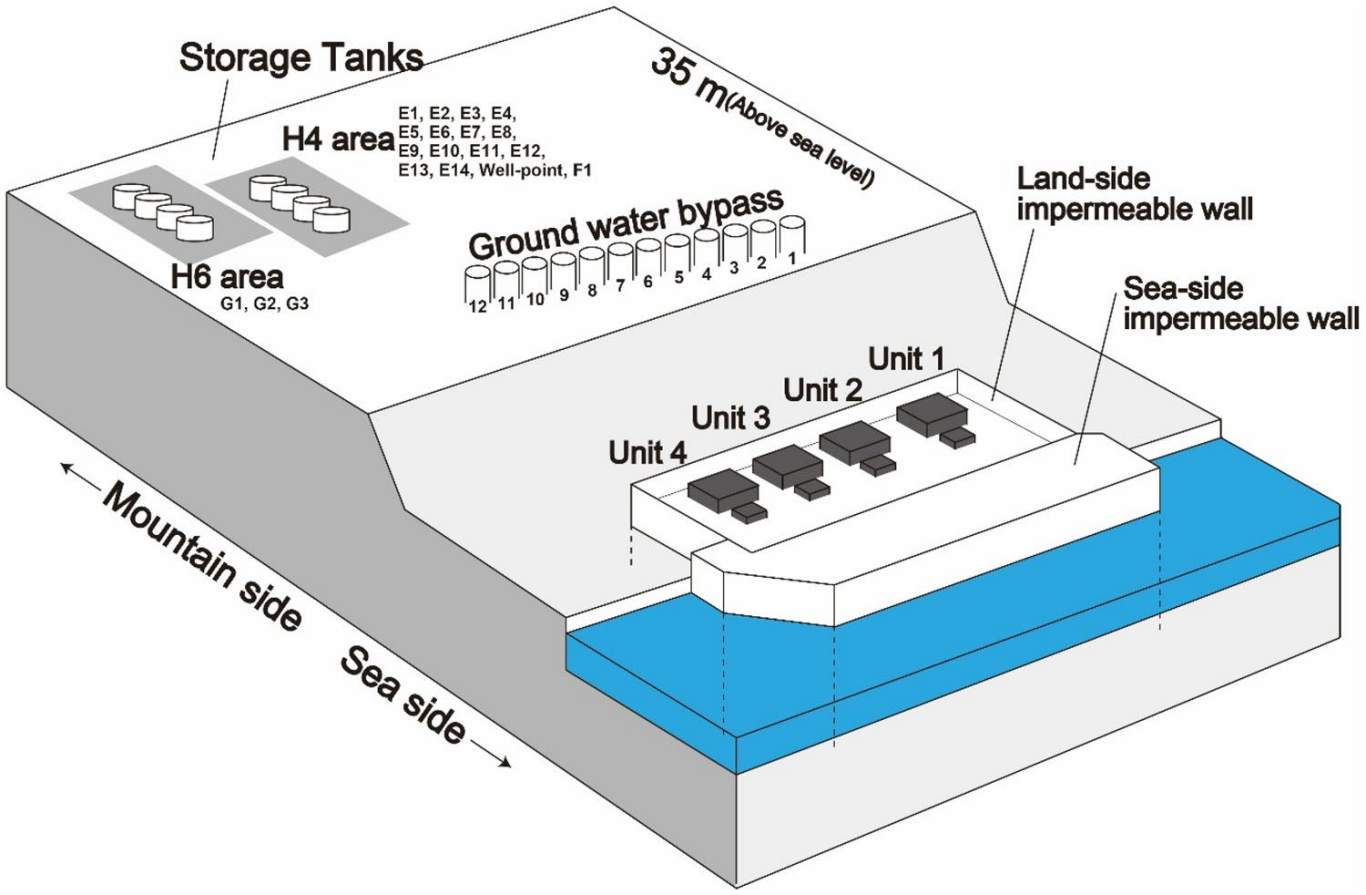
Schematic of FDNPP. The Unit 1 to Unit 4 reactor building and turbine buildings were only 4 m above the north-west Pacific Ocean sea-level.

Figure 1 shows a schematic diagram of the nuclear power plant site after the accident.

The land-side water impermeable wall (frozen soil wall) and the sea-side water impermeable wall (steel sheet pile) were installed to surround the circumference of the FDNPP and prevent ^3^H flow off site. Frozen soil walls block uncontaminated groundwater from getting close to reactors and buildings, while steel sheet piles block potentially contaminated groundwater from spreading into the ocean.

**Figure 2:**
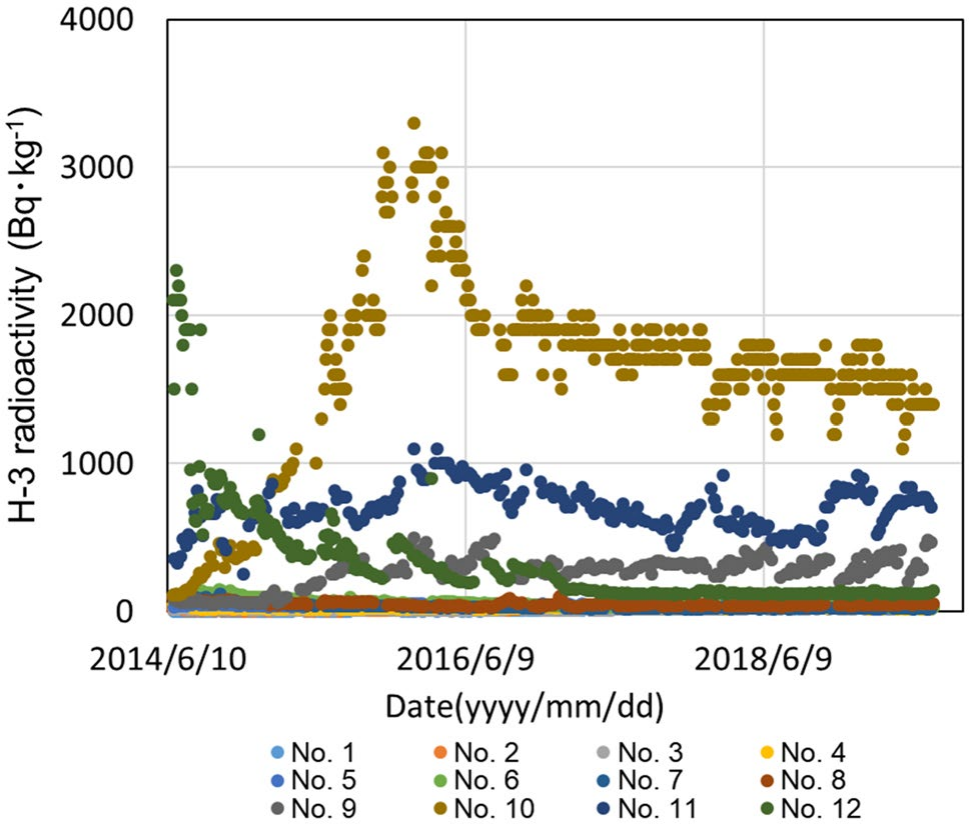
^3^H radioactivity in water collected from wells that facilitate ground water bypass of the FDNPP. The location of wells is shown in Fig. 1.

A series of wells were drilled at 35 m above sea level, upstream of FDNPP, to reduce the amount of groundwater flowing under the reactor building, and the well water was constantly pumped (Ground water bypass). The wells were drilled to a depth directly above an impermeable layer inside the plant's grounds. Figure 2 shows the radioactivity of tritium in groundwater flowing through this bypass from June 2014 to June 2019. The ground water bypass system has 12 wells (No.1 to No.12)^20^, and the highest concentration of radioactivity was in No. 10 well on the south side. The concentration of ^3^H on June 2014 was 10 Bq/L, but it exceeded 3000 Bq/L in April 2016 and has been gradually decreasing since then to approximately 1400 Bq/L in 2019. No. 10 well is next to No.11, which also had levels of ^3^H higher than other wells, at 700 Bq/L as of June 11, 2019. No. 12 is the southernmost well, but unlike No. 10 and No. 11 wells, the tritium levels tended to decrease monotonically from a peak in April 2014^21^.

Groundwater was estimated to flow into the ocean from the mountain side based on ground water flow modeling^22^.

It was not possible to determine from these data whether tritium-contaminated groundwater was still being released as tritium had already spread before the completion of the several barriers. Contaminated water may still be leaking from FDNPP site even after the barrier was completed^23^. The fact that tritium has been continuously detected in groundwater from the bypass installed upstream of FDNPP even after the completion of the water barrier (frozen wall) does not mean that tritium in the groundwater flows to the sea. In addition, the radioactivity trends in the neighboring wells vary widely, indicating that groundwater is moving in a complex manner.

The movement of groundwater may be impacted by the removal of the water from the wells. The amount of water removed from the wells has been changed in a timely manner in order to maintain appropriate groundwater level. If the water level was lowered too much, water flow would be induced from the reactor.

In order to evaluate the absolute amount of tritium contained in well water, information such as flow rate would be required, but TEPCO has not disclosed flow rates publicly.

### ^3^H radioactivity leakage

**Figure 3:**
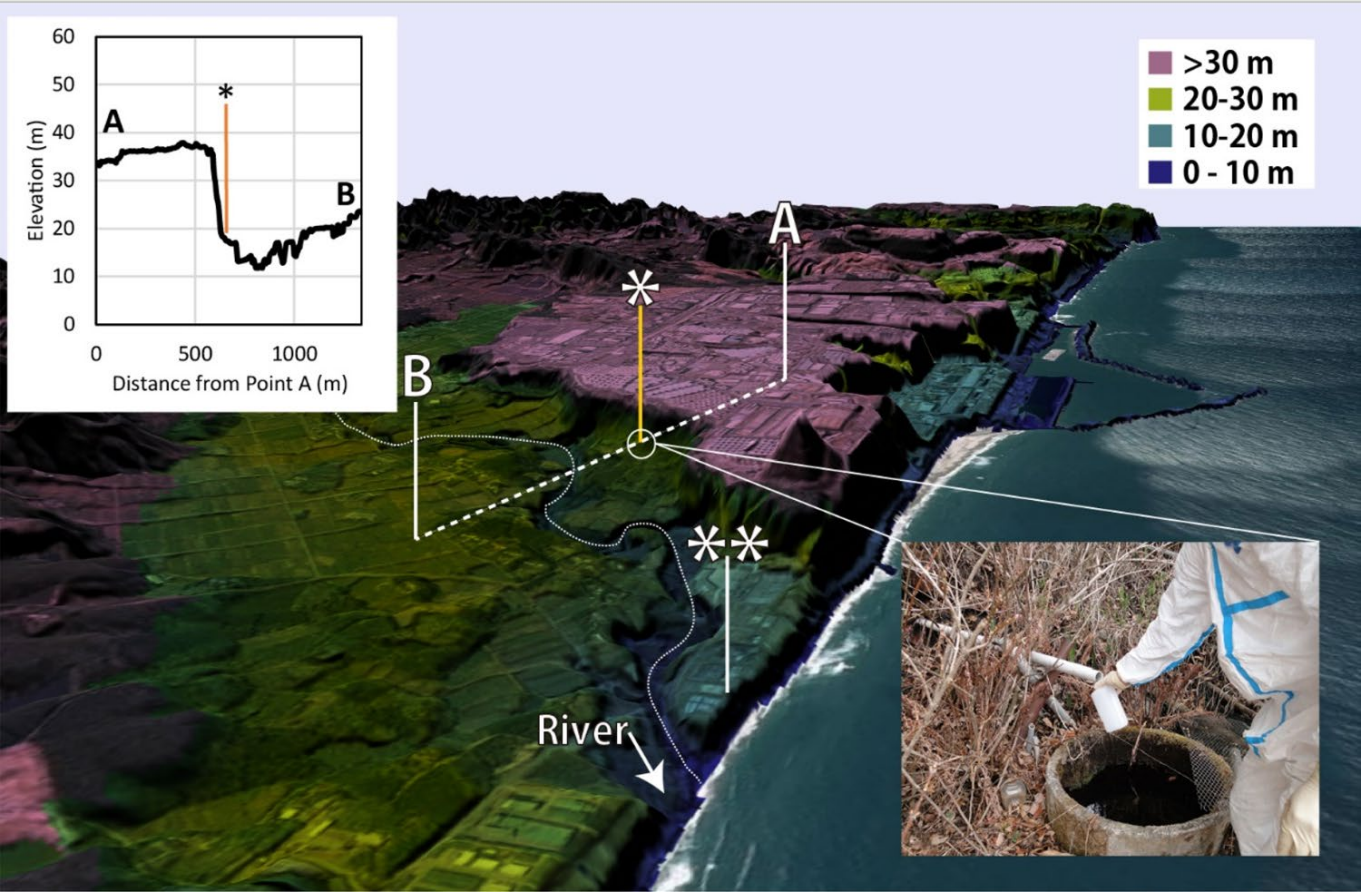
Bird’s eye view of FDNPP from south side. The sump water sampling point was the under the cliff (*), at 17 m above the sea level. The sump water which flowed out from the plastic pipe which stuck in the wall was sampled directly. Sump water was also identified on the shoreline (**). There is a river running on the south side that reaches the north-west Pacific Ocean. This map was created by processing an electronic topographic map by Geospatial information authority of Japan.

**Figure 4:**
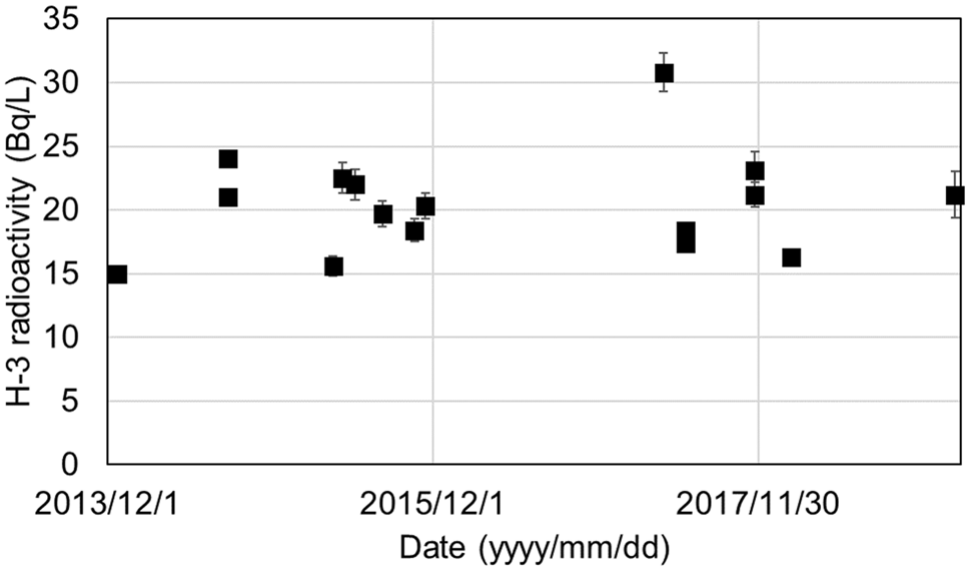
Tritium in sump water collected under the cliff at the boundary of the FDNPP shown in Fig. 3 (single asterisk. *).

The concentration of ^3^H in the sump water collected at the sites indicated by asterisks in Fig. 3 is shown in Fig. 4. The ^3^H observed in sump water ranged from 15 to 31 Bq/L and was almost constant (average 20 Bq/L). The ^3^H exceeded the expected natural level (up to 7.8 TU(1 TU = 0.118 Bq/L), 0.92 Bq/L) of ^3^H, thus it is assumed that the ^3^H originated from FDNPP. Since the sump water were collected directly from cliffs, tritium in sump water would have passed under the ground of FDNPP site.

In addition, the sump water also contained radiocesium (^134^Cs and ^137^Cs). The concentration of ^137^Cs ranged from 3 to 4 Bq/kg, and the ratio of ^134^Cs/^137^Cs radioactivity at the time of the accident was almost 1. This also suggests that the water originated from FDNPP site^24^.

Tritium deposited via the air in surface water is not expected to mix with ground water. No tritium exceeding natural levels was detected in the air and precipitation around the FDNPP during the study period (2013–2019). At the FDNPP, four measures have been taken to prevent surface water from infiltrating into groundwater^25.^.

1. Grouting of surfaces (to prevent from soaking rainwater into the ground) (from Oct. 2014),
2. Pumping of water from the sub-drain (from Sep. 2015),
3. Frozen soil wall around the 4 nuclear plants (from Mar. 2016),
4. Sea-side impermeable wall (from Oct. 2015).

It was clear that there was no direct correlation with the radioactivity of tritium contained in the leachate compared with the respective construction periods.

No tritium above the natural level was detected in the flowing-wells about 500 m away from the nuclear power plant. (see supplementary data).

The flowing-well water tritium concentration ranged from 0.003 to 0.01 Bq/L and was measured using the ingrowth method. Natural level of ^3^H in Japan was ranged from 0.13–0.92 Bq/L. Meanwhile the radioactivity of tritium in flowing water was below 0.01 Bq/L. The radioactivity of the water was at least one eighth. It was considered to be at least three half-lives above conservative estimates. Therefore, it was estimated that the tritium in the groundwater from the flowing-well had an age of nearly 40 years.

## Discussion

### Relationship between countermeasures and contaminated water

Tritium released into the north-west Pacific Ocean by FDNPP as liquid prior to the accident was reported as 2.0 × 10^12^ Bq in 2009, 1.6 × 10^12^ Bq in 2008, 1.4 × 10^12^ Bq in 2007^26^. These tritium releases were legal and planned. Quantitatively, and in comparison, the amount of leakage noted in this research may not be a major problem.

However, it is important to note that there is a route of tritium groundwater leakage on the land-side from the inside to the outside of the site. There may be further leakage in the future. We postulate three scenarios where leakage could occur in the future.

First, there is a possibility that surface water has infiltrated the groundwater. The river water within a 1 km radius around the site in Fig. 3 was sampled and analyzed for ^3^H during the study period, but ^3^H above the natural level was never detected. Therefore, the first possibility was not demonstrated.

**Figure 5:**
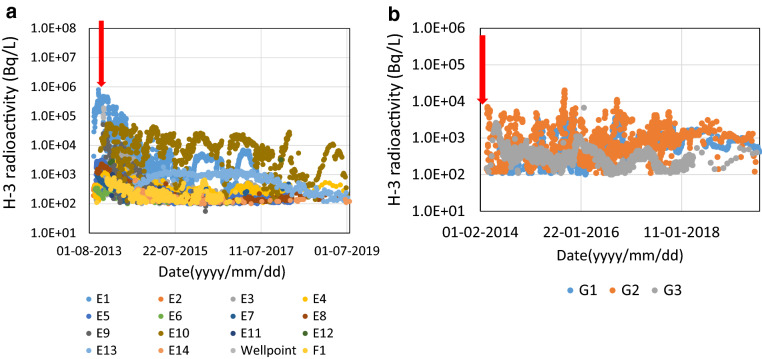
(**a**) Radioactivity of ^3^H in well (E1 to E14, well-point, F1) water around H4 area (inside the plant site). The red arrow indicates when the leak occurred from the tanks. (**b**) Radioactivity of ^3^H in well (G1–G3) water around H6 area (inside the plant site). The red arrow indicates when the leak occurred from the tanks.

Second, there was a possibility of leakage from a group of tanks just above the cliff. In August 2013 in the H4 area and in February 2014 in the H6 area, contaminated water leaked from the tanks to the ground. In the H4 area, outflow was reported to be 8.4 × 10^7^ Bq/L (total beta) over about 300 m^3^, with most of the water infiltrating the ground^27^. In the H6 area, 3.0 × 10^6^–2.3 × 10^8^ Bq/L (total beta) leaked into to about 100 m^3^^28^. The radioactivity of tritium in water pumped from observation wells around the H4 (E1-E14, wellpoint, F1 well) and H6 areas (G1-G3 well) are shown in Fig. 5a,b, respectively. The time of leakage from the contaminated water tank was indicated by the red arrow (Fig. 5a,b). In the H4 area, tritium contained in the E1 well was highest (790 kBq/L, Oct 17^th^, 2013). After 2013, radioactivity in the E1 well-tended to decrease gradually.

A well near the leaking tank (E1, E10) had higher tritium activity than other wells. Therefore, it is possible that tritium leaked from the tank and was continuously mixed into the well water. On the other hand, there was no clear correlation between tritium in wells in the H6 area and leakage. It is possible that the H6 tank leaked water may not have reached the well water in the H6 area yet.

The possibility cannot be ruled out that the contaminated water gradually spread underground and eventually leaked off site. Although no direct evidence of this and of spillage outside the site has been found, there was a possibility that tritium soaked underground due to accidental spills from tanks in 2013 and 2014 and was leaking directly off the site.

Third, groundwater may be spreading horizontally over the impermeable layer below the FDNPP. As shown in Fig. 2 and Fig. 5a,b, tritium has been widely detected in wells at the FDNPP. Therefore, it was reasonable to assume that tritium was seeping from the point where the impermeable layer was cut off by the cliff at the site boundary. However, while tritium radioactivity in the leachate was always constant, there is insufficient data for determining if the tritium is moving along the impermeable layer.

The tritium detected under the cliff was not exceptionally high, but it appears that a hydrologic route has been established for the tritium to leak via underground pathways off site. If a new earthquake or tsunami occurs, more highly contaminated water may flow out though the route. The monitoring system on the land side is very vulnerable and needs to be improved.

### The origin of tritium-containing water

The discussions so far have made it clear that tritium is leaking out of the impermeable layer on the cliffs of the FDNPP. Strontium isotope ratio (^87^Sr/^86^Sr) analysis was carried out to determine whether the water was widely spread on the south side of the plant. A comparison of strontium between water collected at the cliff (* in Fig. 3) and spring water collected at the shoreline (** in Fig. 3) was performed to ascertain if a hydrologic connection between the two sites exists. The results are presented in Table 2. Sr concentrations were not specific at 185 and 121 ppb at the site * and at the site **, respectively. However, when comparing the ^87^Sr/^86^Sr ratios of groundwater at the two sites, the two have distinctly different values over the uncertainty on the Sr isotopic analysis. The difference is more visible if the results are normalized to NIST-987 strontium carbonate isotopic standard (standard value of ^87^Sr/^86^Sr is 0.710248)^29^. For this purpose, the “delta notation” has been applied as per following:

$$\delta (87_{Sr} ) = \left( {\frac{{\left( {\frac{{87_{Sr} }}{{86_{Sr} }}} \right)_{Sample} }}{{\left( {\frac{{87_{Sr} }}{{86_{Sr} }}} \right)_{NIST987} }} - 1} \right) \times 1000$$

**Table 2:** Sr isotopic analysis of ground water between cliff (* in Fig. 3) and shoreline (**).

Sample	Stable Sr *µg/g*	SD *%*	^87^Sr/^86^Sr	SD *%*	δ (^87^Sr)	^n^Sr *Bq/kg*	Detection Limit *mBq/kg*
Cliff	0.185	2.5	0.705948	0.0033	− 6.054	ND	185
Shoreline	0.121	3.1	0.707525	0.0024	− 3.834	ND	120

The obvious divergence of the delta notations reflects distinct water/rock or water/water interactions. Therefore, it is assumed that the hydrogeological origin of groundwater at (*) and (**) are different. This indicates that the flow path of groundwater over impermeable ground layers is not simple. ^90^Sr contamination was not confirmed in both cases, and the ^90^Sr levels were below the detection limit.

Since the end of 2013, tritium originating from the FDNPP has been detected from the south side of the site. This is the first report of continuous tritium detection on the land side of the site. There are 2 possible causes of elevated tritium on the land side of the site: the leakage of contaminated water from the tanks in 2013 and 2014, or the leakage of tritium from the initial accident, which had already spread widely underground at the FDNPP site. The leaking tritium concentration in water was approximately 20 Bq/L, which is lower than the tritium concentration detected at the nuclear site. In addition, ^87^Sr/^86^Sr isotope ratio analysis revealed that groundwater flowing over the impervious layer south of the plant had a different hydrogeological pathway. It appears that an underground route for contaminated water has been established which could lead to future problems. It is necessary to strengthen surveillance of leakage on both the ocean boundary and also land boundary of FDNPP.

## Methods

### Sample collection

**Figure 6:**
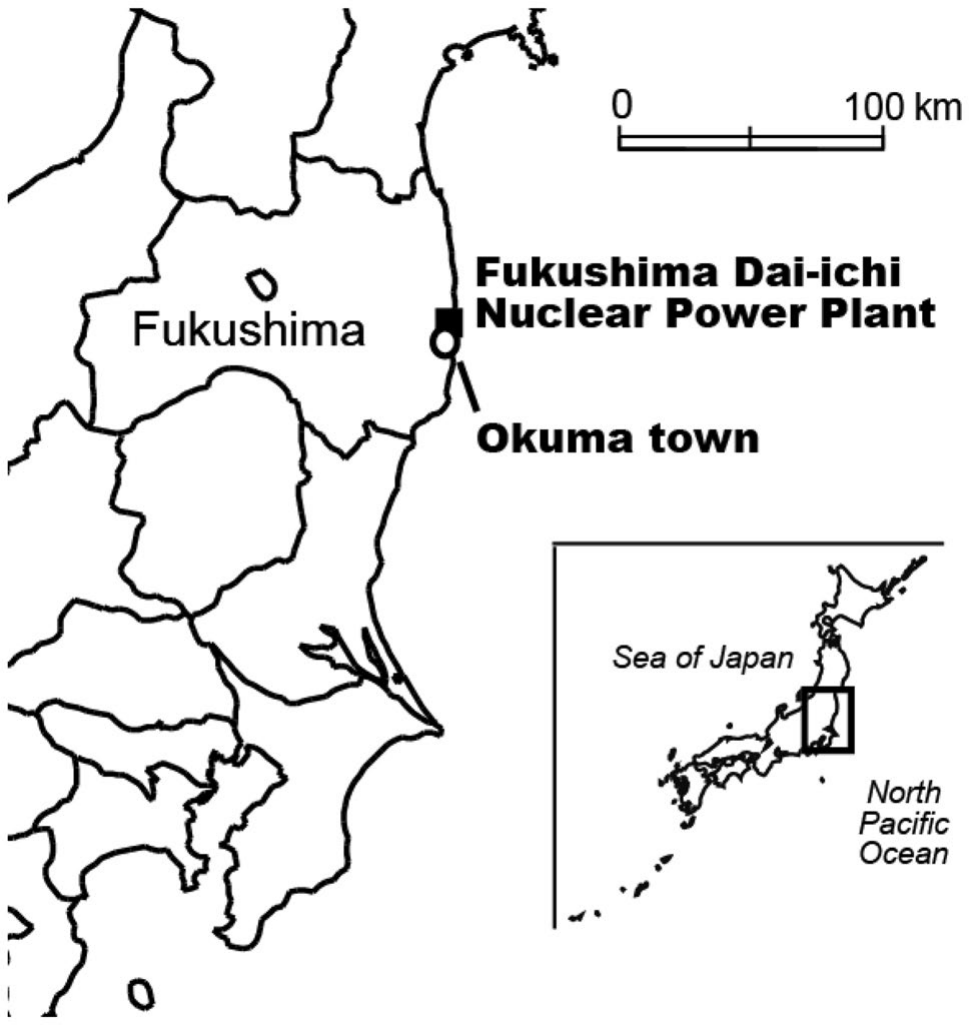
Location of Fukushima Dai-ichi Nuclear power plant (FDNPP), in Fukushima Prefecture, Japan.

Environmental samples were collected in Okuma Town, Fukushima Prefecture, where the FDNPP is located (Fig. 6). The FDNPP is located adjacent to the north-west Pacific Ocean and is 230 km away from Tokyo. Groundwater samples were collected from 2013 to 2019.

Most of Okuma town is still highly contaminated with radioactive cesium, making it difficult for re-habitation. The reactor buildings (R/B) and turbine buildings (T/B) of the FDNPP face the north-west Pacific Ocean at 10 m or less above sea level, as indicated by blue green in Fig. 3. The contaminated water tanks and treatment facilities are shown in pink on a hill at 35 m above sea level (Fig. 3).

Sampling of the sump water was carried out under a cliff at the boundary of the FDNPP at 17 m above sea level. As shown in the photo in Fig. 3, the sump sample was taken directly from a pipe that was stuck into a cliff. Since the pipe was stuck in the cliff wall, it was possible to directly sample the groundwater. Groundwater could be sampled directly from sump without touching contaminated soil or plants, and was the best place to assess groundwater contamination.

### Tritium analysis

^3^H in water was measured by noble gas mass spectroscopy and a liquid scintillation counter. The suspended matter of the environmental water was removed by filtration (Advantec 5C), and ^3^H was extracted according to the official MEXT method^30^. Approximately 50 mg of KMnO_4_ and 50 mg of Na_2_O were added to 50 ml of filtered water and distilled for 1 day.

A liquid scintillation counter (LSC) was used for counting the ^3^H beta emissions (Perkin Elmer Tri-carb 3180 TR). The detection efficiency was determined based on a self-absorption curve with quench correction. The external standard source method using ^133^Ba was applied to the beta count. A 20 mL low potassium LSC vial was used, and 10 mL of the water sample and 10 mL of a cocktail agent (UltimaGold LLT, Perkin Elmer) were added to each vial and stirred well. Beta emissions were measured at least 2 h after stirring to eliminate auto-fluorescence. For almost all samples, amount of quenching i.e. transformed Spectral Index of External Standard (tSIE), was approximately 300–400, with a detection efficiency of approximately 23%.

The measurement time per vial was 1 day, (1-h cycles, 24 times). The ^3^H detection limit was approximately 1.5 Bq/L.

Several samples were measured by noble gas mass spectrometry using the In-growth method to check the ^3^H LSC measurements^3,31^. In the helium-3 ingrowth method, tritium concentrations were measured in the collected samples at AORI, the University of Tokyo, Japan. After filtration, and distillation of the groundwater samples, they were placed into stainless steel bottles. All dissolved gases, including helium, were extracted from the groundwater by pumping and shaking the samples in an ultrasonic bath. The completely degassed water samples were sealed in the containers. Several months later, ^3^He produced by ^3^H decay was analyzed using a conventional noble-gas mass spectrometer at AORI (Helix-SFT; GV Instruments Ltd.). Tritium concentrations were measured indirectly from the measured ^3^He amounts. The detection limit and the analytical uncertainties of the ^3^H measurements were estimated to be approximately 0.003 Bq/L and 10%, respectively. Radioactive decay was corrected for as of the sampling date. Comparison of the ingrowth analytical method with the LSC showed good agreement in measured radioactivity.

### ^87^Sr/^86^Sr and ^90^Sr/^86^Sr isotope analysis

Water collected at two locations (“*” and “**” in Fig. 6) was used for stable Sr isotope ratio (^87^Sr/^86^Sr) analysis and ^90^Sr (T_1/2_ = 28.8y) analysis to determine the origin of the spring water and possible ^90^Sr contamination.

For this purpose, a Phoenix X62 (Isotopx, UK) thermal ionization mass spectrometry (TIMS) was used. For stable strontium concentration measurement, an Agilent-8800 inductively coupled plasma mass spectrometer (Agilent Technologies, USA) was utilized. The detailed strontium analysis method is described elsewhere^32^.

The TIMS is equipped with nine Faraday cups (max 10 V), Daly ion counting system (Photomultiplier PM) (50 mV = 3 × 10^6^ CPS), Secondary electron multiplier (SEM), Rear axial Faraday and four Dynode channel electron multipliers known as Channeltron multiple ion counting system. The collector system also comprises a wide aperture retarding potential (WARP) filter to improve abundance sensitivity and transmission efficiency.

For stable strontium isotopes analysis, multi-dynamic while for ^90^Sr analysis static procedure were applied. ^87^Sr/^86^Sr isotope ratio was measured first and next the ^90^Sr/^86^Sr isotopic ratio.

A 50 mL water sample was evaporated and dissolved with 5 mL 8 M HNO_3_. The HNO_3_ solution was prepared using analytical grade Tamapure AA-100 reagents (68% w/w) and Milli-Q2 purified water (> 18 MΩ cm at 25 °C).

For strontium separation 0.5 mL DGA and Sr extraction chromatography resin of 100–150 µm particle size (Eichrom Technologies Inc, USA) was packed into polypropylene gravity columns (Muromac, Japan; size, 42 mm in length and 5 mm in diameter) and rinsed with 10 mL H_2_O and preconditioned with 5 mL 8 M HNO_3_ with a flow rate of ~ 0.2 mL min^−1^. The sample was dissolved in 5 mL 8 M HNO_3_, loaded into the prepared columns and rinsed with 3 mL 8 M HNO_3_ and 3 mL 3 M HNO_3_. Finally, the Sr was stripped from Sr resin with 3 mL 0.05 M HNO_3_. This final solution was evaporated to about 0.1 mL and mixed with 0.5 ml HNO_3_ and 0.5 mL H_2_O_2_. The solution was boiled for 10 min and evaporated to dryness. Thereafter, the residue containing Sr was dissolved in 0.25 mL 1 M HNO_3_ and evaporated to a tiny drop. This tiny drop containing the Sr was loaded onto a degassed single rhenium filament (99.999%) along with one microliter TaF_5_ activator. More detail of the TIMS analysis is presented elsewhere^33^.

## Supplementary information

Supplementary Information.

## Data Availability

All data supporting the graphs in Figs. 2, 4 and 5a,b are available from the corresponding authors upon request or see supplemental data.
